# Huge chronic expanding hematoma of the iliac bone following multiple hip surgeries: a case report

**DOI:** 10.1186/s13256-018-1783-z

**Published:** 2018-09-04

**Authors:** Masayuki Morishita, Hitomi Hara, Etsuko Katayama, Teruya Kawamoto, Naomasa Fukase, Toshiyuki Takemori, Shuichi Fujiwara, Kotaro Nishida, Ryosuke Kuroda, Toshihiro Akisue

**Affiliations:** 1 Department of Orthopaedic Surgery, Kobe University Graduate School of Medicine, 7-5-1 Kusunoki-cho, Chuo-ku, Kobe, 650-0017 Japan; 2 Division of Orthopaedic Surgery, Kobe University International Clinical Cancer Research Center, 1-5-1 Minatojimaminami-cho, Chuo-ku, Kobe, 650-0047 Japan; 3 Department of Rehabilitation Science, Kobe University Graduate School of Health Sciences, 7-10- 2 Tomogaoka, Suma-ku, Kobe, 654-0142 Japan

**Keywords:** Chronic expanding hematoma, Ilium, Internal hemipelvectomy, Hip transposition, Bone tumor

## Abstract

**Background:**

Chronic expanding hematoma is a rare entity resulting from trauma or surgery. This condition usually occurs in soft tissue, such as the trunk or extremities, while chronic expanding hematoma arising from bone has not been reported previously. We describe an unusual case of a huge intraosseous chronic expanding hematoma arising from the ilium, which had grown over a 40-year period following hip surgeries.

**Case presentation:**

A 57-year-old Japanese woman presented with a 1.5-year history of right hip pain. She had a history of bilateral developmental dysplasia of the hip and had undergone bilateral arthroplasties in childhood. A physical examination revealed a large, firm, immobile mass at her right ilium. Based on radiographic findings, a type of slow-growing bone tumor was suspected, and an incisional biopsy was performed. A histopathologic examination revealed large amounts of old clotted blood within the lesion, and the capsule of the lesion was composed of dense, fibrous, connective tissue. There was no evidence of neoplasia, and chronic expanding hematoma was suspected. The lesion was resistant to conservative treatment, and so we performed an internal hemipelvectomy (including the capsule of the mass) and a reconstruction by hip transposition 2.5 years after the incisional biopsy. There was no recurrence of chronic expanding hematoma at the most recent follow-up of 1 year and 8 months postoperatively.

**Conclusions:**

A chronic expanding hematoma is characterized by its persistence and increasing size more than 1 month after the trauma or surgical event suspected of causing hemorrhage. To the best of our knowledge, this is the first report of chronic expanding hematoma arising from bone. We performed internal hemipelvectomy and hip transposition, and there has so far been no recurrence. This disease may be considered a differential diagnosis for bone tumor when the patient has a history of surgery or trauma, regardless of how many years have passed since the index event.

## Background

Chronic expanding hematoma (CEH) is a rare entity resulting from trauma, surgery, and/or bleeding disorders. Previous reports have described the location of CEHs as the thoracic cavity or the soft tissue of the extremities, while intraosseous CEH has not been reported previously. We describe an unusual case of a huge intraosseous CEH arising from the ilium, which had grown over a 40-year period following hip surgeries. The patient was successfully treated by surgical excision and hip transposition.

## Case presentation

A 57-year-old Japanese woman presented with a 1.5-year history of right hip pain when she walked long distances. She had a history of bilateral developmental dysplasia of the hip and had undergone bilateral acetabular osteotomies in childhood. There was no history of trauma, anticoagulant use, or a collagen vascular disorder. She is a housewife. She has no medical history and family history.

A physical examination revealed a firm, immobile mass measuring 18 cm × 12 cm located on the right side of her ilium. An operation scar measuring 14 cm was found in the front of her hip joint. There was no redness of the skin or swelling of the inguinal lymph nodes. There were no neurological signs of motor or sensory disturbances in her limbs. She could walk with one axillary crutch on one arm and could stand on her right leg. The joint motions of her right hip joint were − 20° extension, 30° flexion, 20° abduction, and 10° adduction. There were no differences in the circumferences of her lower limbs. All laboratory data were within normal limits including coagulation studies: white blood cells (WBC) 7300/uL, hemoglobin 12.8, platelet 22.6 × 10^4^/μL, C-reactive protein (CRP) 0.12 mg/dL, aspartate aminotransferase (AST) 15 U/L, alanine aminotransferase (ALT) 11 U/L, blood urea nitrogen (BUN) 11 mg/dL, creatinine 0.50 mg/dL, activated partial thromboplastin time (APTT) 27.8 seconds, and prothrombin time-international normalized ratio (PT-INR) 0.97 INR).

**Fig. 1:**
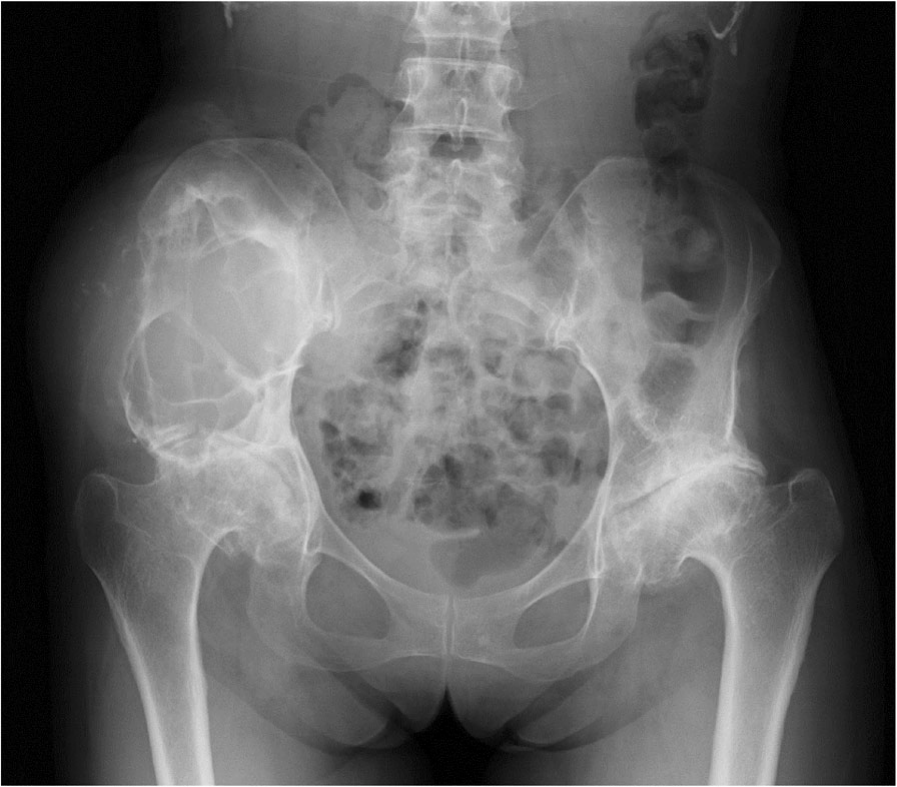
A plain radiograph of the ilium. Initial plain radiography showed marginal sclerosis and calcification inside the bone

**Fig. 2:**
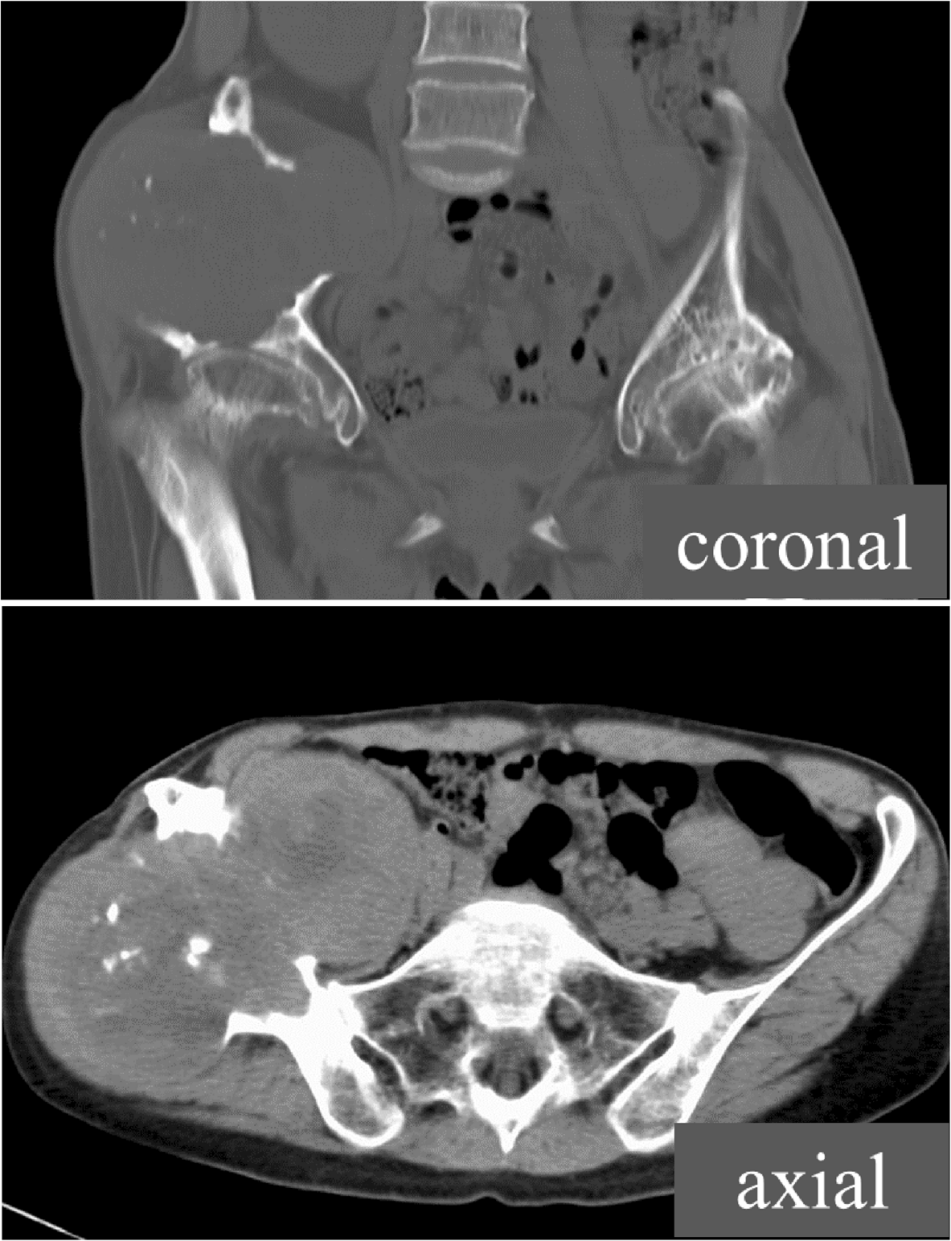
Computed tomography of the ilium. Initial computed tomography demonstrated a heterogeneous mass around the ilium and an area of destroyed bone

**Fig. 3:**
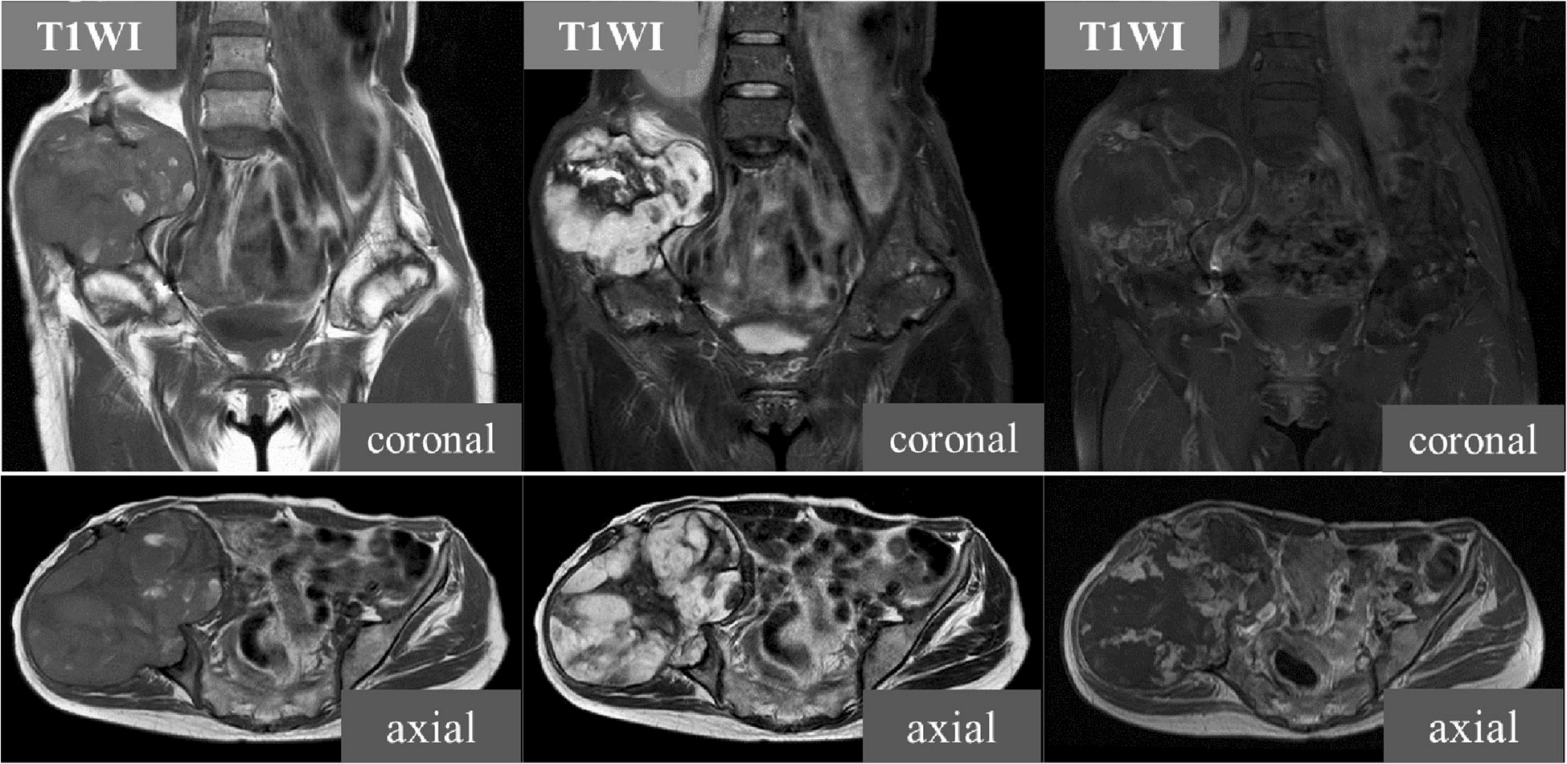
Magnetic resonance imaging of the ilium. The lesion of the right ilium showed predominantly isointense or high signals on T1-weighted images, and a mixture of low and high signal intensities on T2-weighted images. The heterogeneous enhancement of the mass following the intravenous injection of gadolinium-diethylenetriaminepenta-acetic acid is visible on the T1-weighted image

A plain radiograph revealed expanded deformity of her right ilium with marginal sclerosis and calcification inside the bone (Fig. 1). Computed tomography demonstrated a heterogeneous mass around the ilium and an area of destroyed bone (Fig. 2). On magnetic resonance imaging of the same sites, the lesion showed predominantly isointense or high signals on T1-weighted images, and a mixture of low and high signal intensities on T2-weighted images. There was visible heterogeneous enhancement of the mass on a T1-weighted image following the intravenous injection of gadolinium-diethylenetriaminepenta-acetic acid (Gd-DTPA) (Fig. 3).

**Fig. 4:**
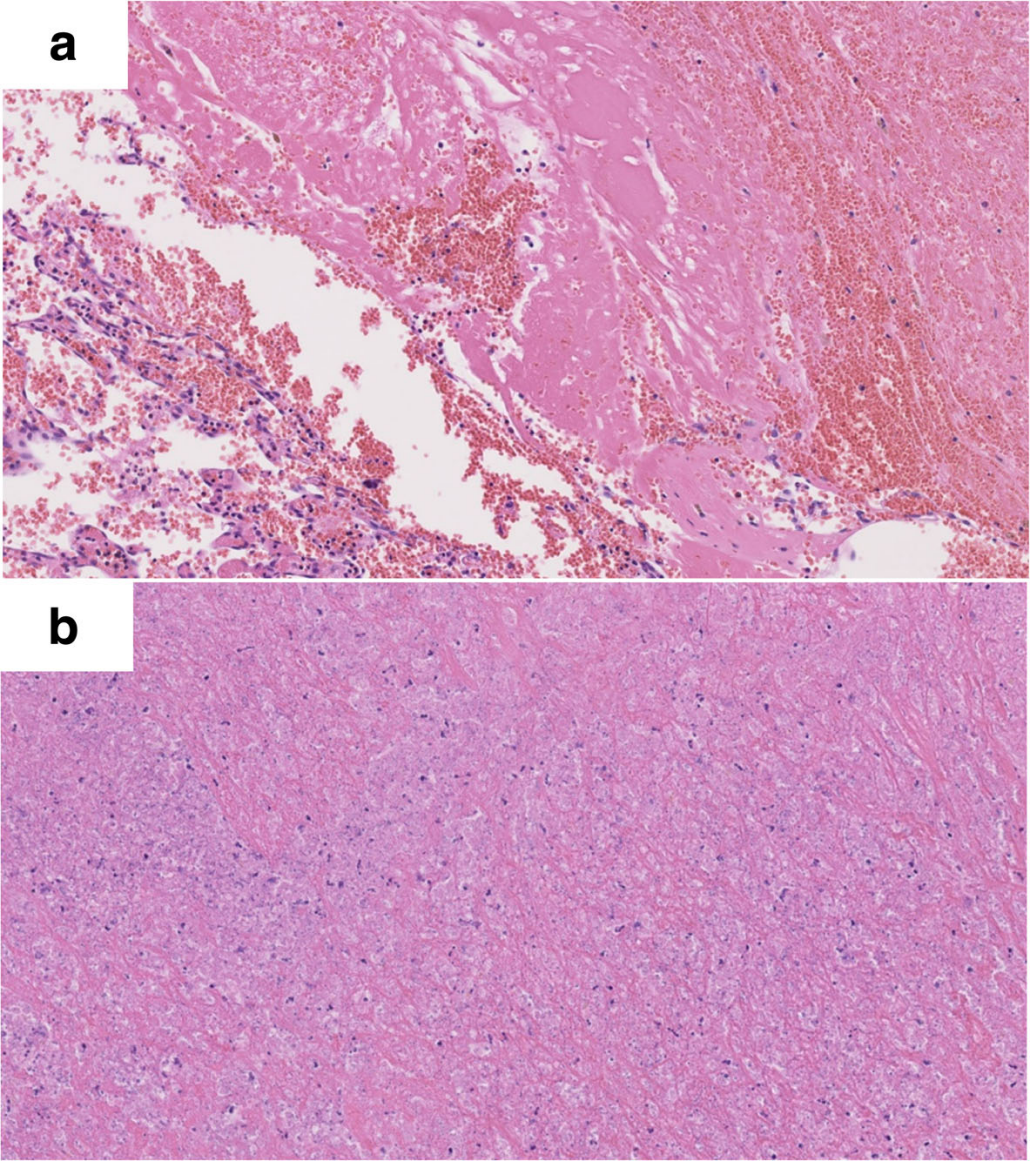
Histopathology of the lesion tissue retrieved in the incisional biopsy. **a** Histopathologic examination demonstrated large amounts of old clotted blood within the lesion. The capsule of the lesion was dense fibrous connective tissue. **b** There was no evidence of neoplasia (hematoxylin and eosin stain)

Based on these findings, our differential diagnoses were giant cell tumor of the bone, aneurysmal bone cyst, or low-grade malignant tumor such as telangiectatic osteosarcoma. Therefore, an incisional biopsy of the lesion was performed. An intraoperative examination revealed that the lesion had a thick capsule; when the capsule was incised, abundant blood was drained from inside. The intraoperative hemorrhage from the incisional biopsy was 500 ml; then, our patient needed a blood transfusion because her hemoglobin level decreased to 6.7 mg/dl from the preoperative level of 12.8 mg/dl. A histopathologic examination revealed large amounts of old clotted blood within the lesion. The capsule of the lesion was composed of dense, fibrous, connective tissue (Fig. 4a). There was no evidence of neoplasia (Fig. 4b). Therefore, CEH was suspected.

**Fig. 5:**
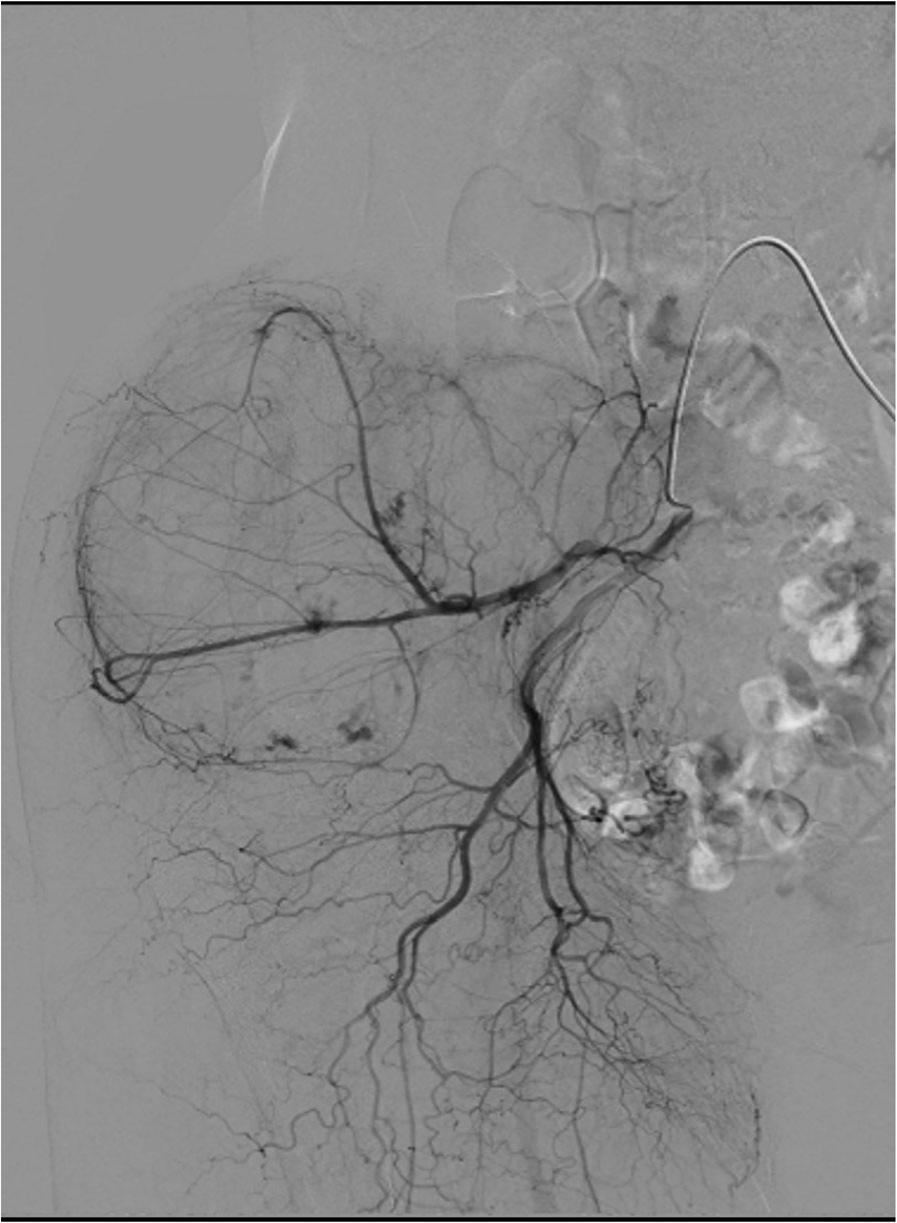
Angiography of the lesion. Angiography showed that some branches of the right internal iliac artery supplied the lesion

**Fig. 6:**
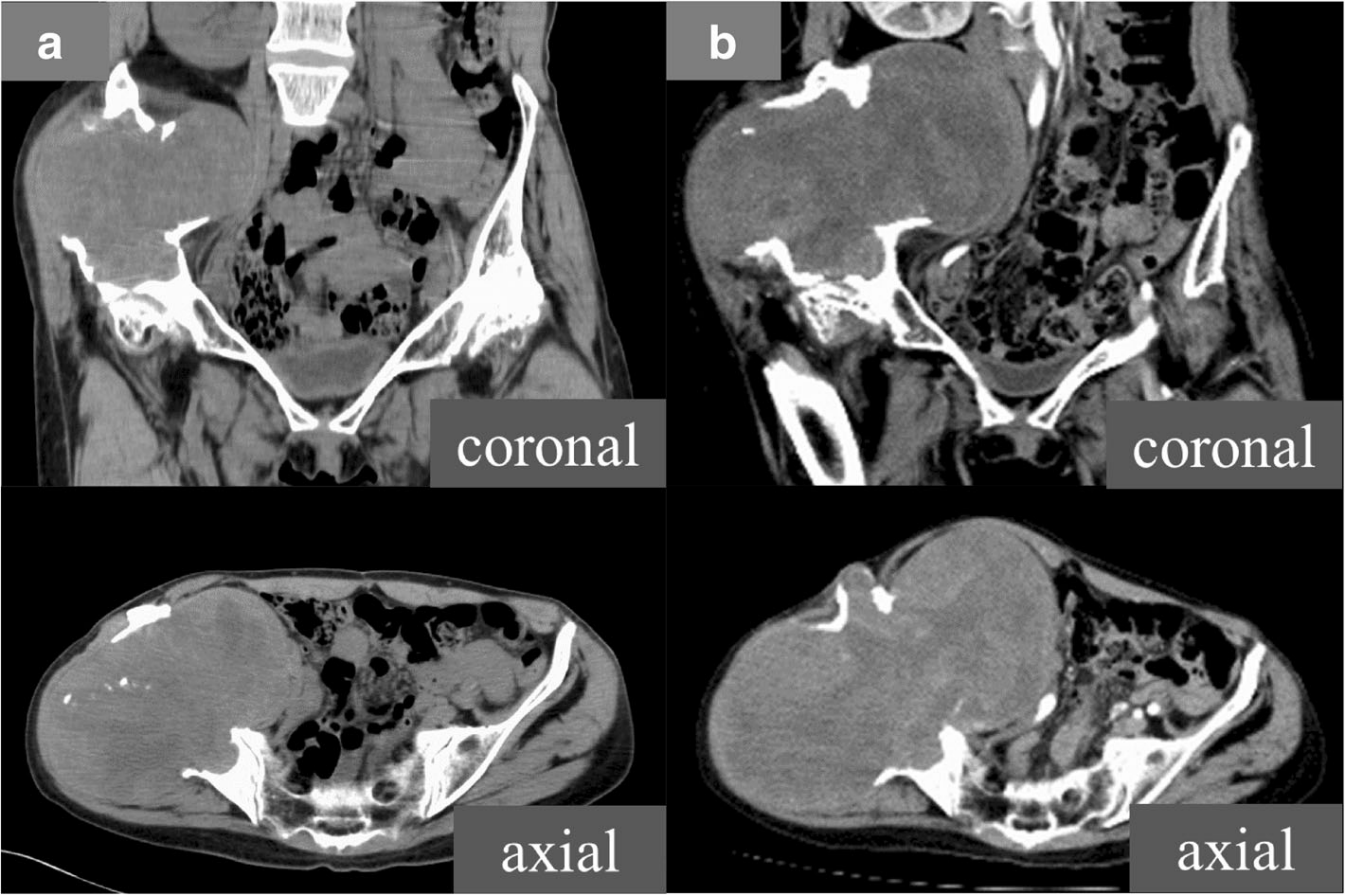
Computed tomography of the ilium at initial presentation (**a**) and 2.5 years after diagnosis (**b**). Although a consecutive selective arterial embolization program was performed, the lesion became significantly larger in 2.5 years

We discussed the treatment options of the current case because there was no previous example of a huge CEH of bone. Surgical treatment was not recommended due to inaccessibility based on our experience of the intraoperative massive hemorrhage at the previous biopsy. We selected non-operative management for the current case. A consecutive selective arterial embolization program was started and performed five times (Fig. 5). In addition, our patient was submitted to an off-label treatment with denosumab, which is a monoclonal antibody and acts as an inhibitor of the RANK/RANKL pathway and diminishes bone turnover. Denosumab was administered using the regimen for giant cell tumors of bone and continued for 3 months. However, the lesion continued to slowly grow, and neuralgia of the femoral nerve occurred (Fig. 6a, b), so we suspected that it might be a malignant bone tumor and decided to perform surgical treatment. We expected that we would be unable to prevent and control the operative bleeding in curettage or volume reduction surgery in this case. Therefore, we performed an internal hemipelvectomy, including the capsule of the mass 2.5 years after the incisional biopsy.

**Fig. 7:**
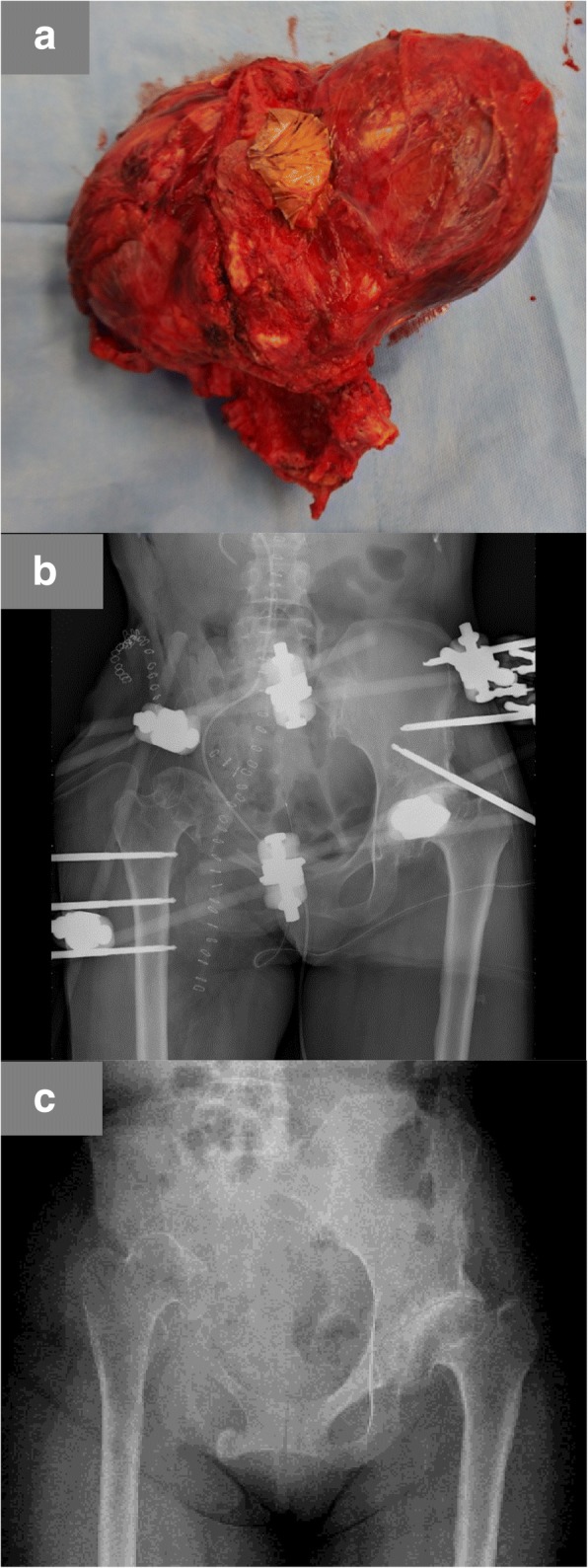
Wide excision of the lesion and hip transposition. **a** The resected specimen. **b** A postoperative plain radiograph with an external fixation apparatus. **c** A plain radiograph taken 1 year postoperatively

At the operation, the mass was completely covered in a capsule, with no evidence of invasion of the neighboring muscle. On macroscopic examination, the lesion was encased in a thick capsule (Fig. 7a). After the lesion was excised, a hip transposition was done as a limb salvage procedure (Fig. 7b). On microscopic examination, the mass was composed of a mixture of fibrin, blood clots, and hemosiderin deposition with a fibrous layer containing degenerated muscle fibers and new capillaries. A histopathologic examination confirmed the diagnosis of CEH consistent with the diagnosis indicated by the incisional biopsy.

External fixation of her pelvis and her right femur was applied for 6 weeks postoperatively. After removing the external fixation, partial weight-bearing was permitted for 4 weeks, and full weight-bearing with one crutch was allowed 14 weeks postoperatively.

There was no recurrence of CEH at the most recent follow-up of 1 year and 8 months postoperatively. She can ambulate with the assistance of one crutch and a heel lift of 5 cm (Fig. 7c).

## Discussion

We described an unusual case of a huge intraosseous CEH arising from the ilium, which had grown over a 40-year period following acetabular osteotomies. Intraosseous CEH has not been reported previously.

CEH was first described by Reid *et al.* as a rare clinicopathological entity characterized by its persistence and increasing size more than 1 month after the trauma or surgical event suspected of causing the hemorrhage [1]. CEH can occur anywhere in the trunk or the musculoskeletal system in the extremities after trauma or surgery [2–4]. However, CEH arising from bone rather than soft tissue is extremely rare.

The detailed mechanism underlying CEH has not yet been clarified. It is thought that the expansion is related to the inflammation stimulated by blood breakdown [5]. These cellular breakdown products then cause a fibroblastic reaction. Inflammation leads to increasing vascular wall permeability and bleeding from dilated capillaries in the granulation tissue beneath the capsular wall, resulting in expansion of the hematoma [6]. Trauma or surgery has been implicated as the cause of many of the reported CEHs [4, 6–9]. In the current case, we consider that prior hip surgeries probably resulted in a gradually enlarging hematoma that moved into the bone marrow space.

CEH can occur at any location in the trunk or extremities, and symptoms differ according to location. Postoperative cases have been reported, most of which have involved the thoracic cavity after surgery of the lung, coronary arteries, or aorta [8, 9], and all the lesions described in previous reports were located in a body cavity or soft tissue. In the current case, the lesion arose from the ilium, which was located in the center of the lesion, and it was unlikely that the lesion arose from soft tissue eroding the iliac bone. To the best of our knowledge, this is the first report of CEH arising from bone.

It is very difficult to differentiate CEH from a malignant tumor, as in both cases the mass slowly expands and occasionally erodes the bone [9]. Magnetic resonance imaging is considered a good diagnostic modality. The findings of central heterogeneity on both T1-weighted and T2-weighted images with a peripheral rim of low signal intensity reflecting fluid collection of fresh and altered blood with a fibrous wall are reportedly more consistent with hematoma than malignancy, and are considered characteristic for differentiation [10]. However, it is sometimes difficult to differentiate hematomas from malignant tumors based on only clinical and radiological findings. In these cases, definitive diagnosis of CEH depends on histopathology. On histopathologic examination, the CEH is well encapsulated and the capsule walls uniformly consist of an outer fibrocollagenous layer and an inner granulation layer [11].

CEH should be managed by complete surgical excision, as incomplete removal may lead to recurrence [12]. In order to achieve complete excision in the current case, we had to perform internal hemipelvectomy and hip transposition as a limb salvage procedure because the lesion had expanded considerably to the acetabulum. The hip transposition procedure is reportedly safe and simple for biological and functional reconstruction of pelvic defects after wide excision of bone sarcoma [13]. Surgical hip transposition involves moving the femoral head proximally to the lateral side of the sacrum or the underside of the resected ilium after resection of the acetabulum. The joint capsule was reconstructed with the use of mesh material, and a pouch was formed for the femoral head. Shortening the leg also allows the acquisition of adequate soft tissue coverage in critical situations, which may be the most important factor for preventing postoperative complications [13]. This method should result in a good clinical course in the current case.

## Conclusions

We have presented an extremely rare case of CEH arising from bone a long period after hip joint surgeries. To the best of our knowledge, this is the first report of CEH arising from bone. We performed internal hemipelvectomy and hip transposition, and there has so far been no recurrence. This disease may be considered a differential diagnosis for bone tumor when the patient has a history of surgery or trauma, regardless of how many years have passed since the index event.

## Abbreviations

ALT: Alanine aminotransferase
APTT: Activated partial thromboplastin time
AST: Aspartate aminotransferase
BUN: Blood urea nitrogen
CEH: Chronic expanding hematoma
CRP: C-reactive protein
Gd-DTPA: Gadolinium-diethylenetriaminepenta-acetic acid
PT-INR: Prothrombin time-international normalized ratio
WBC: White blood cells
